# sIFITM1, sIFITM3, and sViperin antiviral proteins as inactivated CSFV vaccine adjuvants

**DOI:** 10.3389/fvets.2025.1661103

**Published:** 2025-08-26

**Authors:** Shasha Liu, Wenzhuo Zhou, Haobo Ye, Feng Qiu, Rongrong Gu, Erying Xu, Ji-Long Chen

**Affiliations:** ^1^Key Laboratory of Fujian-Taiwan Animal Pathogen Biology, College of Animal Sciences, Fujian Agriculture and Forestry University, Fuzhou, China; ^2^Joint Laboratory of Animal Pathogen Prevention and Control of Fujian-Nepal, College of Animal Sciences, Fujian Agriculture and Forestry University, Fuzhou, China

**Keywords:** IFN-Stimulated Genes (ISGs), antiviral proteins, inactivated CSFV vaccine, vaccine adjuvants, protection efficiency

## Abstract

Vaccine adjuvants are now widely utilized in vaccine formulations. The IFN-Stimulated Genes (ISGs) family, play crucial roles in immune regulation and exhibit broad-spectrum antiviral activity. However, limited studies have investigated the potential of ISGs as vaccine adjuvants. Here, three swine ISGs fusion proteins were induced and purified from *Escherichia coli*, including IFITM1, IFITM3 and Viperin (sIFITM1, sIFITM3, and sViperin). Furthermore, sIFITM1, sIFITM3, and sViperin inhibited the replication of pseudorabies virus (PRV) in swine (PK-15 and 3D4/21) and murine (NIH/3 T3 and C57/B6-L) cells. Importantly, these fusion proteins effectively enhanced the immunogenicity of inactivated classical swine fever virus (CSFV) vaccine and improved the immune response in vaccinated mice. Our evidence indicates that, compared with the CSFV vaccine group, the co-administration of sIFITM1, sIFITM3, and sViperin with CSFV vaccine significantly improved humoral immunity, increased T lymphocyte proliferation in the spleen, and elevated serum IgG antibody levels. In conclusion, this study successfully prepared sIFITM1, sIFITM3, and sViperin fusion proteins, confirming their ability to inhibit PRV replication and suggesting their potential as vaccine adjuvants.

## 1 Introduction

Adjuvants are a class of substances that non-specifically enhance or modulate host specific immune response to antigens. They are typically administered prior to or concurrently with immunogenic agents to promote the induction of a sustained and effective immune response. Adjuvants have become critical components in the formulation of recombinant subunit vaccines (1, 2), protein-toxin vaccines, and inactivated vaccine preparations. Appropriate adjuvant technology can fill several key gaps in the development of modern vaccine products (3, 4). The incorporation of adjuvants markedly decreases the required dose of antigens in vaccination protocols, while also effectively reducing the number of immunizations needed. In addition, certain adjuvants exert immunomodulatory functions by stimulating host to induce the secretion of antibodies belonging to various subtypes. These adjuvants trigger T-cell-mediated immune responses (5, 6), thereby mitigating the potential pathological reactions induced by the antigen itself, enhancing antibody-dependent efficacy, and assisting vaccines in achieving a more durable and effective immune protective effect (7, 8).

Another reason for formulating vaccines with adjuvants is to achieve qualitative regulation of the immune response. Adjuvants can be utilized in preclinical and clinical studies. Additionally, adjuvants functionally elicit an appropriate immune response profile. It has been reported that balanced Th1/Th2/Th17 responses increase the duration of T cell responses and prolong the survival of mice (9, 10). Adjuvants enhance the generation of long-term memory cells (e.g., T-cell memory) (11–13), facilitate the rapid initial response during pandemics (14, 15), and alter the breadth, specificity, or affinity of the immune response (16, 17). Currently, adjuvants are employed to augment the immune response to specific vaccines and reinforce the antibody reaction. These compounds enhance the serum conversion rates in individuals whose reactivity is diminished due to age, disease, or therapeutic intervention. For example, the adjuvant MF59 was used with influenza vaccine to improve response in older individuals (18, 19) and to reduce the dose of vaccine antigen and the number of booster (20, 21).

In recent years, studies have found that there are many ISGs with unique antiviral functions (22). At present, the antiviral spectrum of IFITMs includes more than 20 viruses (23). These viruses include DNA viruses, coated RNA viruses, and non-coated RNA viruses (24–26). Several viruses that are highly pathogenic in humans are suppressed by IFITMs, including human immunodeficiency virus (HIV) (27), Ebola virus (EBOV) (28), Influenza A virus (IAV) (29, 30), Zika virus (ZIKV) (31), and Severe acute respiratory syndrome corona virus (SARS-CoV) (32). Among drugs with antiviral activity, IFITM3 has the best effect (33).

At present, the comprehensive antiviral mechanism of IFITM mainly includes IFITM-mediated inhibition of virus fusion into plasma membrane, lysosome or endosome membrane, so as to achieve the purpose of inhibiting virus entry, rather than restricting virus entry through specific recognition of virus components (33). IFITMs also regulate the pH of endosomes or lysosomes (34). The conformation of some viral envelope proteins (such as hemagglutinin) changes at low endosomal pH, which mediates semi-fusion of the virus and endosomal membrane (35). In addition, IFITM3 inhibit the replication of some non-enveloped viruses by modulating the function of late endosomes. IFITMs reduce the infectivity of some newly created viruses. For example, IFITMs co-locate with HIV-1 Env and Gag and become part of newborn virions, inhibiting the entry of virions into new host cells (27). Recently, IFITMs have been found to inhibit HIV-1 protein synthesis, thereby limiting viral infection (36).

*In vitro* antiviral activity of Viperin has been shown to work against a variety of viruses (37). Viperin blocks the release of IAV particles from infected cells by inhibiting an enzyme in the mevalonate pathway, farnesyl diphosphate synthetase (FPPS) (38), suggesting that this metabolic pathway plays a role in viral excretion. High expression of Viperin occurs in Respiratory syncytial virus (RSV)-infected macrophages that are not allowed to be cultured with RSV (39, 40). Although Viperin shows anti-RSV activity *in vivo* (41), its mode of antiviral action has not been established. Additionally, Viperin has antiviral effects against Human cytomegalovirus (HCMV) for the first time. Overexpression of Viperin in human fibroblasts prior to HCMV infection significantly decreased the expression of late viral proteins such as gB, pp28, and pp651 (42). So, Viperin has anti-broad viral activity against DNA and RNA viruses. Viperin can regulate cellular lipid metabolism during HCMV infection (43). The precise mechanisms by which Viperin exerts its antiviral effects warrant further investigation.

While most studies on IFITMs and Viperin have focused on their function in host innate immunity, this study focuses on the function of IFITMs and Viperin as vaccine adjuvants. Swine IFITM1, IFITM3, and Viperin fusion proteins were prepared as adjuvators and immunized with classical swine fever virus (CSFV) E2 protein recombinant baculovirus inactivated vaccine. To explore its ability to enhance the immune protection effect of CSFV inactivated vaccine, and provide a new method for better control and prevention of CSFV.

## 2 Materials and methods

### 2.1 Cell lines and cell culture

Swine kidney cell line PK-15, swine alveolar macrophage cell line 3D4/21, mouse embryonic cell line NIH/3 T3, and mouse lung fibroblast cell line C57/B6-L were purchased from American Type Culture Collection (ATCC). The above cell lines were maintained in complete Dulbecco’s modified Eagle’s medium (DMEM) supplemented with 10% fetal bovine serum (FBS) and 100 U/mlpenicillin-streptomycin (Beyotime Biotechnology, Shanghai, China). All cells were incubated at 37°C with 5% CO_2_.

### 2.2 Expression plasmid construction

PK-15 cells were used for total RNA extraction with the total RNA extraction Kit (Foregene, China). One microgram of total RNA was used for cDNA synthesis with M5 Single-tube qPCR RT kit (Mei5 Biotechnology, China). Primers sequences used are shown in Table 1. The 393 bp of swine IFITM1, 456 bp of swine IFITM3, and 1,107 bp of swine Viperin gene was excised from the gel and purified using DNA purification kit.

**Table 1:** Primer sequences used for PCR.

Primer name	Primer sequence (5′ → 3′)
IFITM1 (swine) forward	CCGCTCGAGATGGATCAAGAGCCAG
IFITM1 (swine) reverse	GCATCTAGACTAGTAGCCTCTGTTACTCTT
IFITM3 (swine) forward	CCGCTCGAGATGAACTGCGCTTCC
IFITM3 (swine) reverse	GCATCTAGACTAGTAGCCTCTGTAATCCTT
Viperin (swine) forward	CCGCTCGAGATGTGGACACTGGTA
Viperin (swine) reverse	GCATCTAGATCACCAGTCCAGCTTCAG

The purified DNA segments of swine IFITM1, IFITM3, Viperin and the expression vector pCold-TF were digested with XhoI and XbaI restriction enzymes. The purified DNA of IFITM1, IFITM3, and Viperin were cloned into pCold-TF expression vector. There is a thrombin cleavage site between the pCold-TF chaperone protein and ISGs (the recognition sequence is LVPRGS, which cuts between R and G, leaving a residual GS sequence). The pCold-TF vector is a cold-shock inducible expression vector designed to fuse and express the soluble tag known as “Trigger Factor (TF) companion”, which is a prokaryotic ribosome-associated chaperone protein and can facilitate the co-translational folding of newly synthesized polypeptides. The protein size expressed by pCold-TF vector is approximately 48 kDa. The recombinant pCold-TF-IFITM1, pCold-TF-IFITM3, and pCold-TF-Viperin were transformed into the *Escherichia coli* (*E. coli*) DH5a-competent cells; the positive clones were selected and determined by restriction enzyme analysis with XhoI and XbaI.

### 2.3 Recombinant protein expression and purification

The recombinant plasmids pCold-TF-IFITM1, pCold-TF-IFITM3, and pCold-TF-Viperin were transformed into *E. coli* DH5a-competent cells, they were coated on solid LB petri dishes containing ampicillin and cultured at 37°C for 14 h. Positive single colonies were selected from the above petri dishes and inoculated into liquid medium containing antibiotics, and cultured at 37°C and 200 rpm until OD600 was 0.8–1.0, then isopropyl-*β*-d-thiogalactoside (IPTG) was added into the bacterial solution, and the final concentration of IPTG in the bacterial solution was 0.5 mmol/L. Subsequently, the bacterial solution containing IPTG was induced and cultured in a constant temperature shaking table at 16°C and 200 rpm for 16 h. Harvested *E. coli* were lysed by sonication and subjected to 10% sodium dodecyl sulfate polyacrylamide gel electrophoresis (SDS-PAGE). The recombinant proteins were separately purified by Ni-NTA or ammonium sulfate.

### 2.4 SDS-PAGE and Western blotting

The collected cell samples were subjected to cell lysis using Beyotime (Shanghai, ST506). The protein concentration was quantified using the BCA Protein Assay Kit (Beyotime, Shanghai, P0012). Subsequently, the protein samples were separated by SDS-PAGE. For Western blotting, the next step after SDS-PAGE was to transfer to NC membranes (Merck Millipore, HATF00010) and detected with antibodies as indicated. PRV-gE was presented from Chen’ lab and actin was purchased from Proteintech Group, Inc. (Cat. 66,009-1-Ig).

### 2.5 Viruses and viral infection

The swine influenza virus H3N2 was propagated in specific-pathogen-free (SPF) chicken embryos. Pseudorabies virus (PRV) was propagated in PK-15 cells. H3N2 and PRV were used to infect PK-15, 3D4/21, NIH/3 T3, and C57/B6-L cells. Cells were incubated with virus for 1 h and cultured in DMEM for the indicated times, as described previously (44).

### 2.6 Endotoxin determination assay

Bacterial endotoxin was quantitatively detected by Chromogenic LAL Endotoxin Assay Kit (Beyotime, China). Simply was as follows: First, diluted the endotoxin standard solution to the specified concentration with endotoxin detection water. Subsequently, took the endotoxin-free centrifuge tube and added endotoxin detection water, endotoxin standard solution or the sample. Then added the endotoxin detection reagent solution and incubated at 37°C in the dark for 7 min. Added the chromogenic reagent solution and incubated at 37°C for 10 min. Then, added the reaction solutions A, B, and C, and measured the absorbance value at a wavelength of 545 nm. Finally, a standard curve was established: Y = bX + a, and the endotoxin content of each sample was calculated.

### 2.7 Bicinchoninic acid assay

The Bicinchoninic Acid (BCA) assay mainly consists of the following two steps. The first step is to draw the standard curve. Simply is as follows: Dilute BSA by multiple ratios as per the instructions. Add the protein standard solution diluted in a certain ratio to the standard wells. The absorbance was measured at a wavelength of 562 nm and a standard curve was plotted.

The second step is to detected purified protein concentration. Simply is as follows: The sample is added to the BCA working solution and incubated at 37°C for 30 min. The absorbance at A562 nm was determined by an enzyme-linked immunosorbent assay (ELISA) reader, and the protein concentration of the sample to be tested was calculated according to the standard curve.

### 2.8 RNA preparation, RT-PCR, and RT-qPCR

Total RNA was isolated using TRIzol reagent (TIANGEN, China) and reverse transcribed into cDNA utilizing M-MLV Reverse Transcriptase (Promega, United States). The cDNA was analyzed by RT-PCR using Taq DNA polymerase (GenStar, Beijing, China) and by RT-qPCR using SYBR Green Master Mix (Vazyme, Nanjing, China). Primers used are shown in Table 2. *β*-actin was chosen as a reference housekeeping gene for internal standardisation. The data of RT-qPCR analysis were shown in normalized ratios which was auto-calculated using the 2^−ΔΔCt^ method, as described previously (45).

**Table 2:** Primer sequences used for RT-PCR and RT-qPCR.

Primer name	Primer sequence (5′ → 3′)
β-actin (swine) forward	CGGCATCCACGAAACTACCT
β-actin (swine) reverse	GCCGTGATCTCCTTCTGCAT
PRV-gE forward	TTTGGATCCATGCGGCCCTTTCTG
PRV-gE reverse	TTTGAATTCTTACGACACGGCGTCGCA
H3N2-NP forward	CCACAAGAGGGGTCCAGATT
H3N2-NP reverse	GGAGATTTCGCTGCACTGAG
β-actin (mus) forward	GCTGCCTCAACACCTCAACCC
β-actin (mus) reverse	GTCCCTCACCCTCCCAAAAG
IFN-β (mus) forward	ATGAGTGGTGGTTGCAGGC
IFN-β (mus) reverse	ACCTTTCAAATGCAGTAGATTCA
IL-28 (mus) forward	CCATCGAGAAGAGGCTGCTT
IL-28 (mus) reverse	GTCTGCAGCTGGGAGTGAAT
IL-6 (mus) forward	TCCGGAGAGGAGACTTCACA
IL-6 (mus) reverse	GTCTTGGTCCTTAGCCACTCC

### 2.9 Plaque assay

After PK-15 cells were infected with PRV for 2 h, the adjuvant was subsequently added and incubated for 24 h. Then, the culture supernatant was collected for plaque assay. Specifically, MDCK cells were inoculated with the serially diluted supernatant for 1 h. Following this, the cells were washed three times with PBS and overlaid with medium containing methylcellulose. After incubation at 37°C for 72 h, the plaques were quantified.

### 2.10 Grouping and immunization of experimental animals

CSFV E2 protein recombinant baculovirus vaccine, inactivated (Strain WH-09) was a veterinary drug available over the counter. Its primary component was the CSFV E2 protein and it was purchased from Wuhan Keqian Biology Co., Ltd. 75 female BALB/c mice were randomly divided into His-tagged pCold-TF chaperone protein group (control), normal saline + CSFV vaccine group (Vaccine), sIFITM1 protein + CSFV vaccine group (sIFITM1 + Vaccine), sIFITM3 protein + CSFV vaccine group (sIFITM3 + Vaccine), sViperin protein + CSFV vaccine group (sViperin+Vaccine). The fusion protein (dissolved in PBS, 30 μg/mouse) is mixed with CSFV vaccine. On the 1st day, the mixture of vaccine and the adjuvant are injected into the thigh muscle. The mice were re-immunized with the mixture on the 21th day, and were euthanized and sampled on the 28th day (or the 7th day after the secondary immunization).

### 2.11 Organ coefficient measurement

Spleen and thymus tissues of mice were collected, and the blood on the surface was drained with filter paper, then weighed and calculated. The specific formula is as follows: Spleen coefficient = spleen wet weight (mg)/mouse body weight (g); Thymus coefficient = thymus wet weight (mg)/mouse body weight (g).

### 2.12 Extraction and culture of splenic lymphocytes

The spleens of mice were dissociated using a cell strainer to obtain a single-cell suspension. Following the addition of a separation solution and subsequent centrifugation, a distinct layer of circular, milky-white lymphocytes was harvested. Subsequently, a complete culture medium composed of RPMI-1640 supplemented with 10% fetal bovine serum (FBS) was added to prepare a splenic lymphocyte suspension at a concentration of 1 × 10^6^ cells/mL.

### 2.13 Splenic T lymphocyte proliferation rate

The prepared mouse splenic lymphocyte suspension was added to 96-well cell plate, the blank control hole was added with RPMI-1640. Concanavalin A (ConA, a plant hemagglutinin and has a good promoting effect on T lymphocyte transformation) (Sigma-Aldrich, United States) with a final concentration of 5 μg/mL was added and cultured in a 5% CO_2_ incubator at 37°C for 24 h. Subsequently, cell proliferation was detected by the CCK8 method, as follows: Add 10 μL of CCK8 (Sigma-Aldrich, United States) to each well and continue the culture for 2 h. OD values were measured by enzyme-linked immunoassay at 450 nm wave length, and lymphocyte stimulation index (SI) = (experimental hole OD − blank hole OD)/(control hole OD − blank hole OD) × 100%.

### 2.14 Enzyme-linked immunosorbent assay

After enucleation of the eyeballs of mice and collection of blood samples, the blood was left to stand at room temperature for 1 h, centrifuged at 1000 rpm for 20 min, and the serum was collected and frozen at −20°C according to the single usage. Then, the protein levels of IgG or CSFV E2 antibody were examined by enzyme-linked immunosorbent assay (ELISA) using the mouse IgG analysis kit (Jiangsu Enzyme Exemption Industry Co., Ltd.) or CSFV E2 analysis kit (Amoy Lunchangshuo Biotech, Co., Ltd.) according to the manufacturer’s instructions.

### 2.15 Histopathological analysis

BALB/c mice were sacrificed and dissected on the 7th day after the secondary immunization. Mice spleen and thymus were collected and fixed in 4% paraformaldehyde and embedded with paraffin. Then, 4-mm-thick sections were prepared and stained with hematoxylin and eosin (H&E). The slides were visualized under an Olympus BH-2 microscope (Tokyo, Japan).

### 2.16 Statistical analysis

The data were analyzed by One-way ANOVA using GraphPad Prism 5 statistical software. For RT-qPCR analysis, the folding changes of mRNA expression and the normalized domestic genes were determined by technical methods. The SDS-PAGE assay of the three fusion proteins were quantified by ImageJ software to detect the protein purity. Statistical comparisons between groups were performed using Student’s *t*-test. Data are presented as the mean ± SD (standard deviation). A *p*-value < 0.05 was considered statistically significant.

## 3 Results

### 3.1 Prokaryotic expression and purification of sIFITM1, sIFITM3, and sViperin proteins

#### 3.1.1 Induced expression and solubility analysis of sIFITM1, sIFITM3, and sViperin

To better express soluble proteins in prokaryotic cells, we employed pCold-TF vector to construct recombinant plasmids pCold-TF-IFITM1, pCold-TF-IFITM3, and pCold-TF-Viperin. The pCold-TF vector encodes a chaperone protein that facilitates the folding of newly synthesized polypeptides. Subsequently, we transformed *E. coli* with the constructed recombinant plasmids pCold-TF-IFITM1, pCold-TF-IFITM3 and pCold-TF-Viperin. Induction of expression was carried out by adding 0.1 mM IPTG, adjusting the oscillation conditions to 16°C, and incubating at 200 rpm for 16 h. The expression of recombinant proteins were detected by SDS-PAGE analysis. Swine IFITM1 (sIFITM1), swine IFITM1 (sIFITM3), and swine Viperin (sViperin) proteins were successfully expressed, and their bands were close to 66 kDa, 69 kDa, and 93 kDa, respectively, consistent with the expected size (Supplementary Figures S1A–C). The results indicated that the recombinant proteins sIFITM1, sIFITM3, and sViperin were successfully induced.

**Figure 1:**
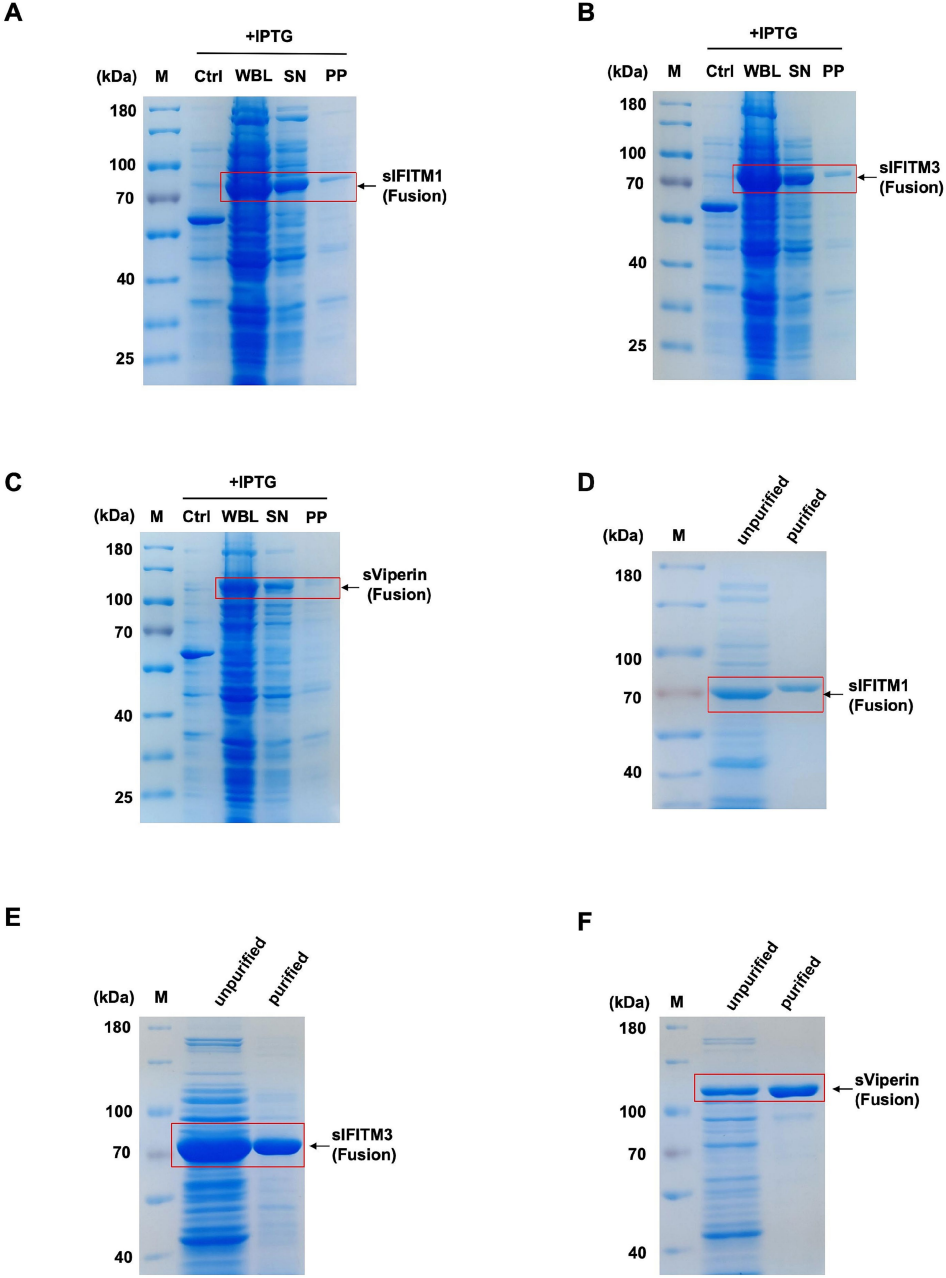
Prokaryotic expression and purification of sIFITM1, sIFITM3, and sViperin proteins. **(A–C)** The solubility analysis of fusion proteins sIFITM1, sIFITM3, and sViperin was determined by SDS-PAGE. Solubility of sIFITM1 **(A)**, sIFITM3 **(B)**, and sViperin **(C)**. M, marker; WBL, whole bacterial lysate; SN, supernatant; PP, precipitate. **(D–F)** Fusion proteins sIFITM1, sIFITM3, and sViperin were purified by Ni-NTA affinity chromatography, and the purification degree of fusion proteins was determined by SDS-PAGE. Purification of sIFITM1 **(D)**, sIFITM3 **(E)**, and sViperin **(F)**.

Further, SDS-PAGE was utilized to detect the solubility of recombinant proteins. The results showed that recombinant proteins sIFITM1, sIFITM3, and sViperin were mainly expressed in the supernatant (Figures 1A–C), indicating that most of the sIFITM1, sIFITM3, and sViperin are fusion proteins.

#### 3.1.2 Purification of sIFITM1, sIFITM3, and sViperin

Next, the fusion proteins sIFITM1, sIFITM3, and sViperin were purified by Ni-NTA affinity chromatography and eluted with 500 mmol/L imidazole. The data showed that relatively fusion proteins sIFITM1, sIFITM3, and sViperin were obtained after Ni-NTA purification (Figures 1D–F).

**Figure 2:**
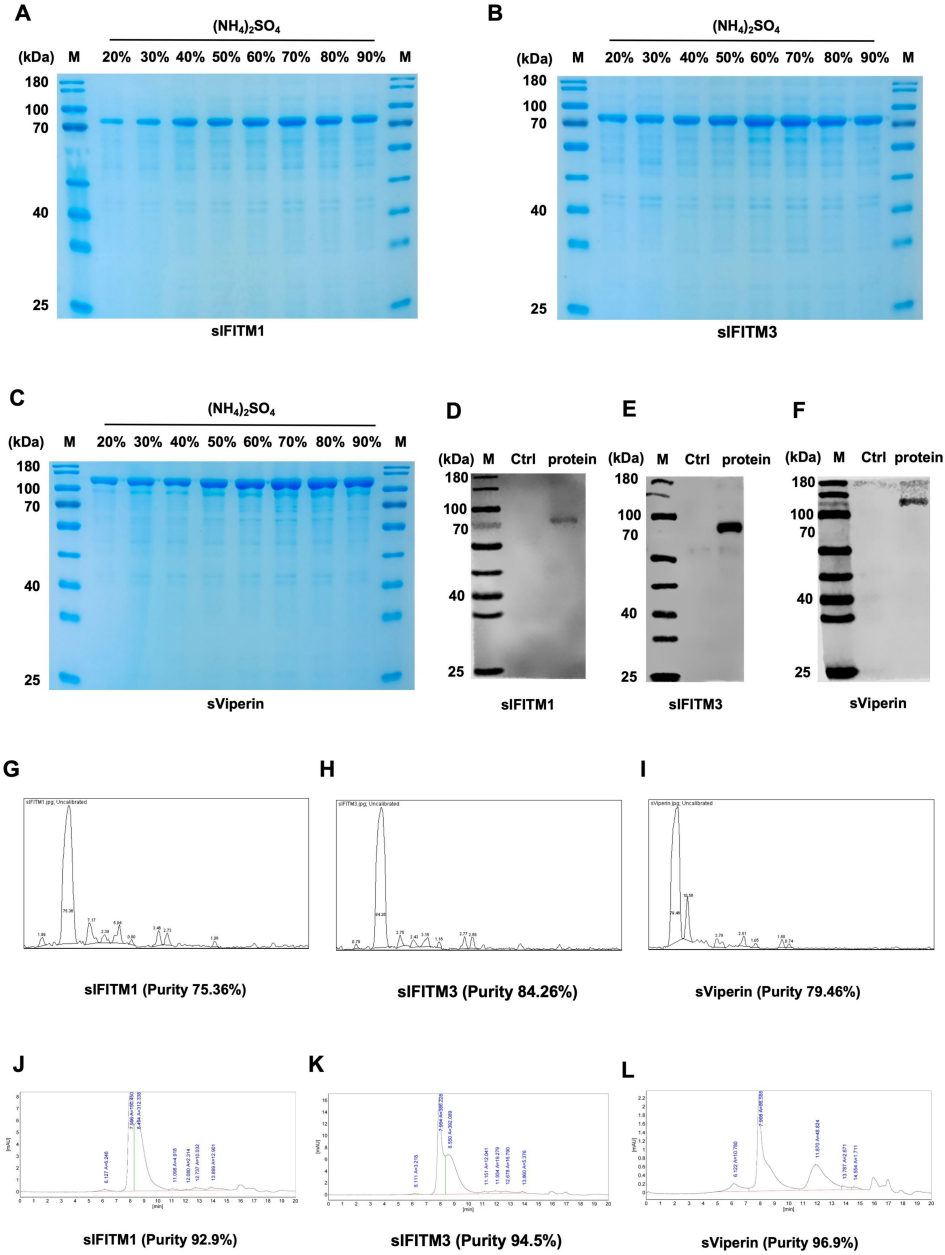
The fusion proteins were obtained by ammonium sulfate precipitation. **(A–C)** Fusion proteins sIFITM1, sIFITM3, and sViperin were purified by ammonium sulfate precipitation method, and the purification degree of fusion proteins was detected by SDS-PAGE. Purification of sIFITM1 **(A)**, sIFITM3 **(B)**, and sViperin **(C)**. **(D–I)** His-tag was utilized to analyze the expression of fusion proteins sIFITM1 **(D)**, sIFITM3 **(E)**, and sViperin **(F)**. And quantitative analysis was conducted using ImageJ software **(G–I)**. **(J–L)** Protein purity of fusion proteins sIFITM1 **(J)**, sIFITM3 **(K)**, and sViperin **(L)** was detected by HPLC.

Moreover, since we consider the practical production applications of these three fusion proteins in the future, we explored more efficient and convenient purification methods, such as ammonium sulfate precipitation. This method is simple to operate, can be precipitated several times to purify the degree, and has a wide range of applications. Fusion proteins sIFITM1, sIFITM3, and sViperin with high purity could be obtained (Figures 2A–C). In addition, we determined the optimal ammonium sulfate saturation for fusion protein purification. The results showed that the fusion protein sIFITM1, sIFITM3, and sViperin could achieve good purification effect when the saturation of ammonium sulfate was 70% (Figure 2A), 60% (Figure 2B), and 70% (Figure 2C), respectively.

Next, we tested the purity of the three fusion proteins. Since the constructed pCold-TF-IFITM1, pCold-TF-IFITM3, and pCold-TF-Viperin recombinant plasmid carried His-tag, anti-His was used as the primary antibody to detect the size of the purified target protein. The results by SDS-PAGE assay showed that there was a single specific band at the molecular weight of 66 kDa (sIFITM1, Figure 2D), 69 kDa (sIFITM3, Figure 2E), and 93 kDa (sViperin, Figure 2F). The quantitative analysis indicated that the protein purity of sIFITM1 is 75.36%, that of sIFITM3 is 84.26%, and that of sViperin is 79.46% (Figures 2G–I). The preliminary experiments show that the fusion proteins were successfully purified, and the purity was good. Further, we performed high performance liquid chromatography (HPLC) assay to purify and quantify the fusion proteins. The purity of the fusion proteins were as follows: 92.9% for sIFITM1 (Figure 2J; Supplementary Table S1), 94.5% for sIFITM3 (Figure 2K; Supplementary Table S2), and 96.9% for sViperin (Figure 2L; Supplementary Table S3). Moreover, the BCA assay indicated the concentrations of the fusion proteins were as follows: 3470 μg/mL with a total volume 0.8 mL and a purification efficiency of 27.76 mg/L for sIFITM1; 5,300 μg/mL with a total volume 0.8 mL and a purification efficiency of 42.40 mg/L for sIFITM3; 6,020 μg/mL with a total volume 0.8 mL and a purification efficiency of 48.16 mg/L for sViperin. In addition, the endotoxin was 1.1028 EU/mL for sIFITM1, 1.0727 EU/mL for sIFITM3, 1.0575 EU/mL for sViperin, respectively, which was originally lower than the national regulation of no more than 50 EU/mL. The above results indicate that the fusion proteins obtained by the ammonium sulfate precipitation method are sufficient to serve as adjuvants for subsequent experiments. Therefore, the fusion proteins sIFITM1, sIFITM3, and sViperin obtained by the ammonium sulfate precipitation were adopted in the following experiments.

### 3.2 Effects of sIFITM1, sIFITM3, and sViperin on the replication of viruses

#### 3.2.1 Effect of sIFITM1, sIFITM3, and sViperin on innate immune response in swine cells

**Figure 3:**
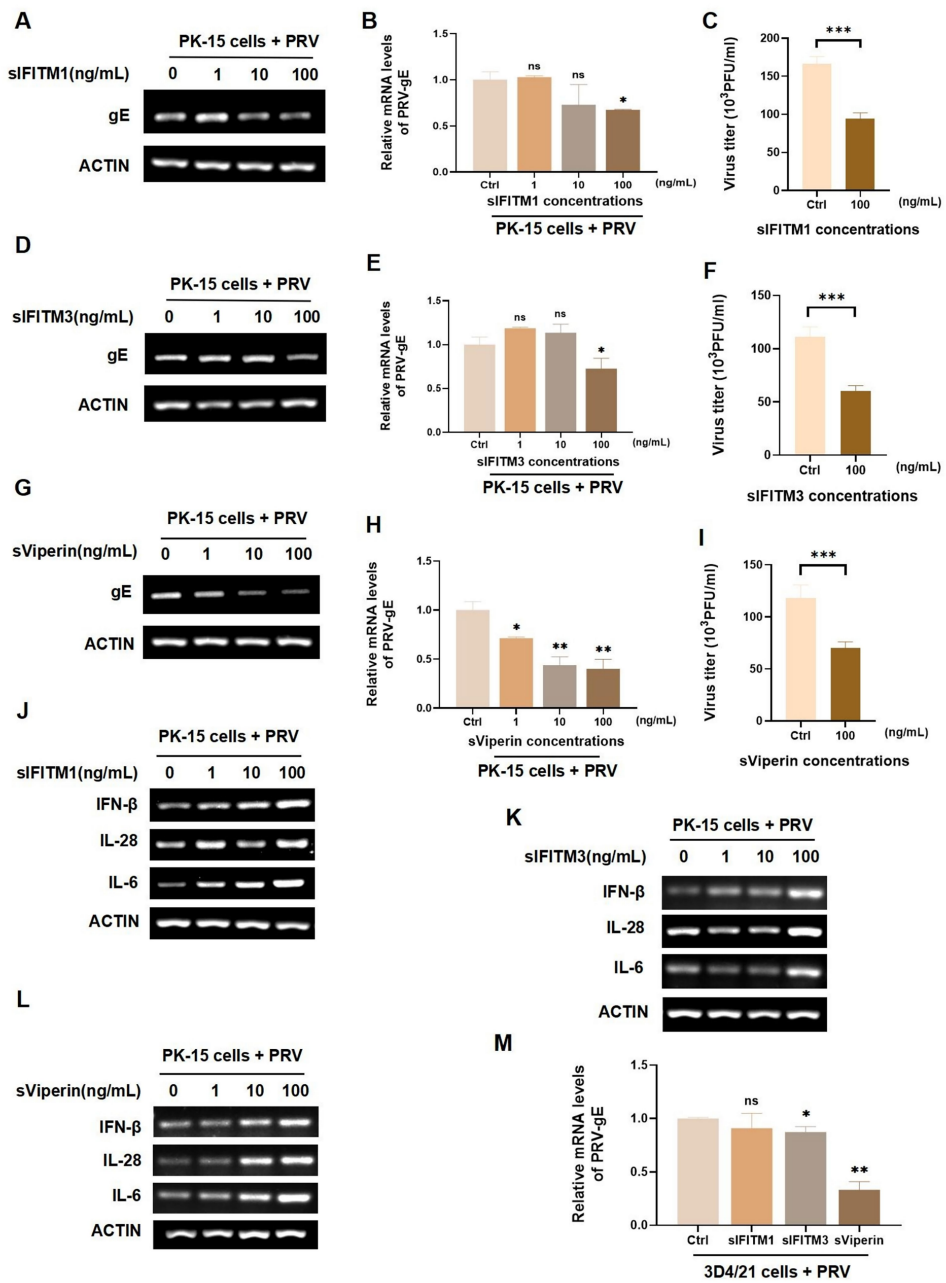
Analyze the effect of sIFITM1, sIFITM3, and sViperin on innate immune response in swine cells. **(A–L)** After adsorption of PK-15 cells by PRV (MOI = 1) for 2 h, fusion proteins sIFITM1 (A-C, J), sIFITM3 (D-F, K) and sViperin **(G–I,L)** were added to PK-15 cells for 24 h at the indicated concentrations, respectively. The replication of PRV in PK-15 cells was detected by RT-PCR **(A,D,G)** and RT-qPCR **(B,E,H)**. Viral titers of PRV in the supernatant were detected by plaque assay **(C,F,I)**. The mRNA level of indicated cytokines was detected by RT-qPCR (J-K). Data are represented as mean ± SD. NS, no significant; * *p* < 0.05; ** *p* < 0.01. **(M)** Following a 2 h adsorption period of PRV (MOI = 1) on 3D4/21 cells, the fusion proteins sIFITM1, sIFITM3, and sViperin were introduced to cells at concentrations of 100 ng/mL respectively, for 24 h. Subsequently, RT-qPCR assays were conducted to assess the viral replication efficiency. Data are represented as mean ± SD. NS, no significant difference, * *p* < 0.05, ** *p* < 0.01.

PRV is a neurophilic virus that can infect a variety of animals, including vertebrates, carnivores, and rodents (46, 47). Swine PRV have been reported in many countries around the world, which has caused great economic losses. According to literature reports, IFITM1, IFITM3, and Viperin have antiviral effects (23, 48). In order to investigate whether the fusion proteins sIFITM1, sIFITM3, and sViperin purified in this study have anti-PRV effects, swine kidney cells PK-15 were infected with PRV (MOI = 1) for 2 h. Then, the different concentrations of fusion proteins (1 ng/mL, 10 ng/mL and 100 ng/mL) were added for 24 h. The replication of PRV was detected by RT-PCR and RT-qPCR. The data showed that the mRNA levels of PRV-gE in sIFITM1, sIFITM3, and sViperin treatment groups was significantly reduced in a dose-dependent manner (Figures 3A,B,D,E,G,H). When the concentration of fusion protein reached 100 ng/mL, sIFITM1 and sIFITM3 could inhibit the mRNA levels of PRV-gE (Figures 3A,B,D,E,G,H), and also inhibit the virus titers of PRV (Figures 3C,F,I). And sViperin could significantly damaged the PRV-gE mRNA expression (Figures 3G,H). To further explore the impact of these fusion proteins on the innate immunity, we detected the expression of several portal cytokines related to innate immunity, such as IFN-*β*, IL-6, and IL-28. The data indicated that sIFITM3 and sViperin remarkably promoted the expression of IFN-β, IL-6, and IL-28 in PRV-infected PK-15 cells (Figures 3J–L). In addition, sIFITM3 and sViperin remarkably inhibited the PRV-gE expression in swine 3D4/21 cells (Figure 3M).

To further explore the antiviral function of sIFITM1, sIFITM3, and sViperin, we infected PK-15 cells with swine influenza H3N2 for 2 h. sIFITM1, sIFITM3, and sViperin with indicated concentration were incubated for 24 h to detect the replication of H3N2 in PK-15 cells. And the NP of H3N2 in sIFITM1, sIFITM3, and sViperin treatment groups was extremely inhibited at the mRNA levels (Supplementary Figures S2A–C). Moreover, we found that sIFITM1 can effectively inhibit protein levels of H3N2 in PK-15-infected cell (Supplementary Figure S2D). Importantly, sIFITM1, sIFITM3, and sViperin can effectively inhibit the viral titers H3N2 in PK-15-infected cell (Supplementary Figures S2E–G). Together, these results indicated that the fusion protein sViperin remarkably inhibit PRV and H3N2 replication in swine PK-15 and 3D4/21 cells.

#### 3.2.2 Effect of sIFITM1, sIFITM3, and sViperin on the replication of PRV in murine cells

**Figure 4:**
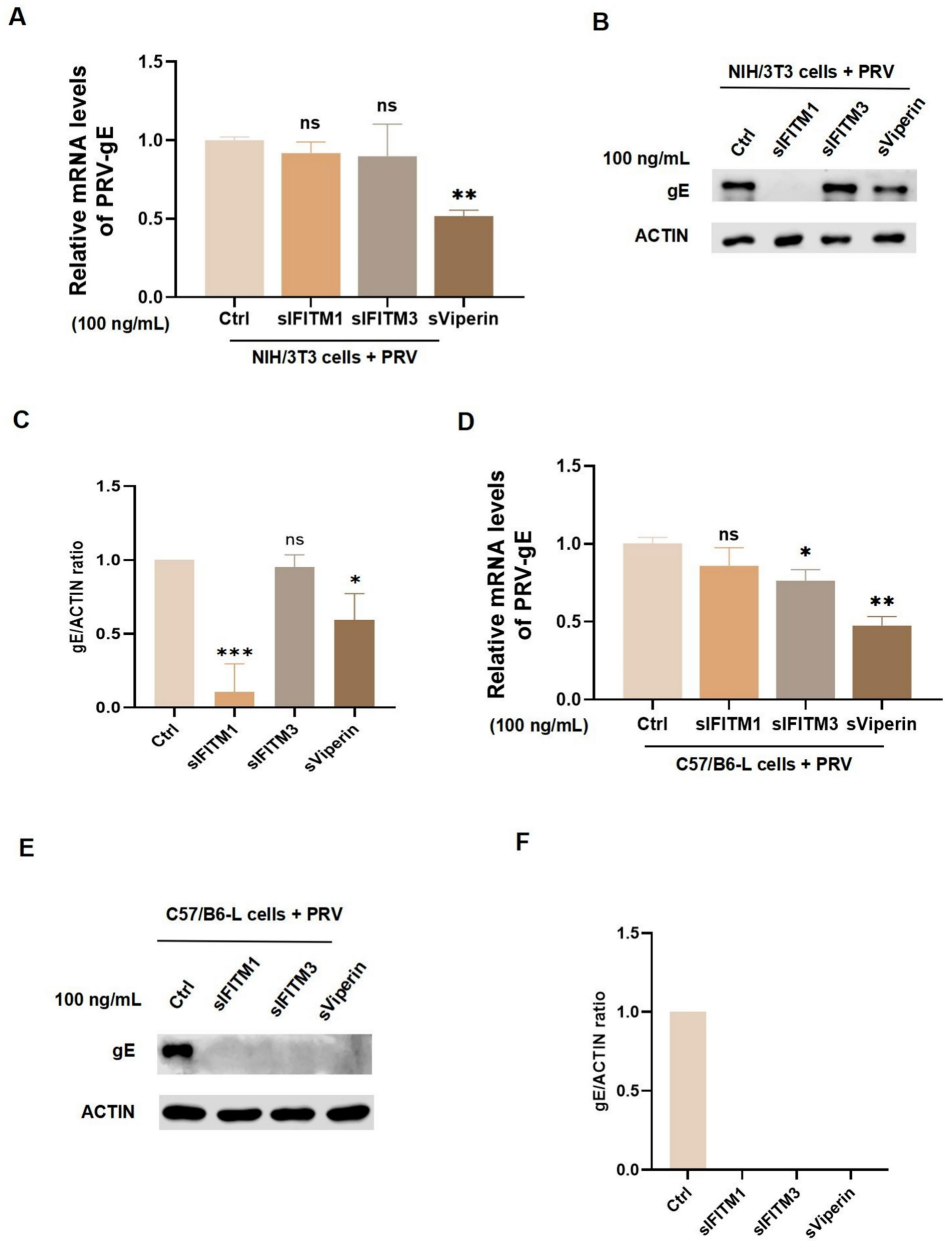
Detect the effect of fusion proteins on the replication of PRV in murine cells. **(A–D)** After a 2 h adsorption period of NIH/3 T3 cells **(A,B)** and C57/B6-L cells **(C,D)** with PRV (MOI = 1), fusion proteins sIFITM1, sIFITM3, and sViperin were added at the concentration of 100 ng/mL for 24 h. The replication efficacy of PRV-gE was assessed by RT-qPCR **(A,C)**. Immunoblots were performed to examine expression of gE **(B,E)**. The gE levels were quantitated by densitometry and normalized to ACTIN levels **(C,F)**. Data are represented as mean ± SD. NS, no significant; * *p* < 0.05; ** *p* < 0.01; *** *p* < 0.001.

Since the homology of the genes of the three antiviral proteins involved in this study was high in mice and swines: IFITM1 was 77%, IFITM3 was 75%, and Viperin was 83%. Therefore, we infected murine NIH/3 T3 cells and C57/B6-L cells with PRV and incubated them with 100 ng/mL sIFITM1, sIFITM3, and sViperin to further explore their antiviral functions. In NIH/3 T3 cells, sViperin was found to significantly inhibit the mRNA expression of PRV-gE, whereas sIFITM1 and sIFITM3 exhibited no notable inhibitory effect (Figure 4A). Interestingly, sIFITM1 and sViperin significantly reduced the protein levels of PRV-gE in NIH/3 T3 cells (Figures 4B,C). In C57/B6-L cells, both sIFITM3 and sViperin were both capable of suppressing the mRNA expression of PRV-gE, while the effect of sViperin is more significant (Figure 4D). sIFITM1, sIFITM3, and sViperin inhibit the protein levels of PRV-gE in C57/B6-L cells (Figures 4E,F). Collectively, these results indicated that the inhibitory effects of the three adjuvants on the protein levels of PRV-gE are more significant than those on the mRNA levels.

### 3.3 Efficacy evaluation of adjuvants sIFITM1, sIFITM3 and sViperin for inactivated swine fever vaccine

#### 3.3.1 Adjuvants sIFITM1, sIFITM3, and sViperin promote the proliferation of spleen lymphocytes in immunized animals

**Figure 5:**
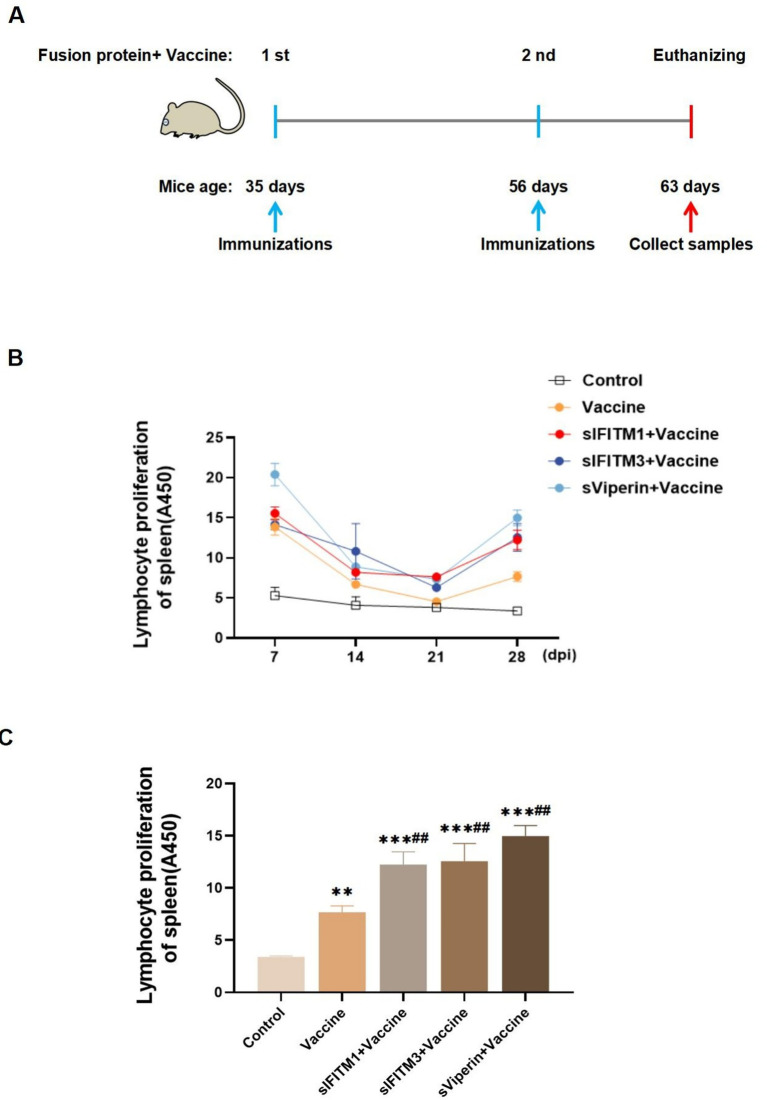
Fusion proteins as adjuvants promote the proliferation of spleen T lymphocytes in immunized mice. **(A)** Shown is a paradigm of the mice immunization program. **(B)** During the immunization period, 3 mice in each group were randomly euthanized every 7 days, spleen lymphocytes were isolated, and the proliferation rate of spleen T lymphocytes was detected. **(C)** On the 7th day after the secondary immunization, 3 mice in each group were randomly euthanized, spleen lymphocytes were isolated, and the proliferation rate of spleen T lymphocytes was detected. Compared with the control group, * means *p* < 0.05, ** means *p* < 0.01, *** means *p* < 0.001; Compared with the vaccine group, # means *p* < 0.05 and ## means *p* < 0.01.

Purified sIFITM1, sIFITM3, and sViperin were utilized as adjuvants together with CSFV E2 protein recombinant baculovirus vaccine, inactivated (Strain WH-09) vaccine to immunize BALB/c mice (0.1 mL/mouse). The fusion protein (dissolved in PBS, 30 μg/mouse) is mixed with the inactivated CSFV vaccine. On the 1st day, the mixture of vaccine and the adjuvant are injected into the thigh muscle. The mice were re-immunized with the mixture on the 21th day, and were euthanized and sampled on the 28th day (or the 7th day after the secondary immunization) (Figure 5A). During the immunization period, the spleens of mice were isolated to a single-cell suspension. Following centrifugation, the second layer of ring-shaped, milky white cells were harvested and identified as lymphocytes. Then lymphocytes were stimulated with ConA to promote T lymphocyte transformation and proliferation rate were detected. The data showed that the spleen T lymphocytes proliferation rate of mice with fusion protein group was higher than that of vaccine group and His-tagged pCold-TF chaperone protein (control) group (Figure 5B). On the 7th day after the secondary immunization, the spleen T lymphocyte proliferation rate in all groups was extremely significantly increased compared with the control group or vaccine group (*p* < 0.01), among which the sViperin+ Vaccine group had the greatest increase (Figure 5C).

In order to have a deeper understanding of this change in immune status, we further tested the spleen and thymus coefficient. Compared with the vaccine group, the spleen coefficient of sIFITM1, sIFITM3, and sViperin groups were significantly increased (*p* < 0.01), while the thymus coefficient was not significantly different (*p* > 0.05) (Table 3). This suggests that sIFITM1, sIFITM3, and sViperin fusion proteins can affect the spleen coefficient of immune mice, but have no significant effect on the thymus coefficient of immune mice. Together, these results indicate that sIFITM1, sIFITM3, and sViperin can promote the proliferation of spleen lymphocytes in immunized mice, and sViperin has the best effect.

**Table 3:** The effects of fusion proteins sIFITM1, sIFITM3 and sViperin on the organ coefficients of immunized mice.

Group	Ingredient	Spleen coefficient	Thymus Index
A	Control	5.11 ± 0.27	1.27 ± 0.54
B	Vaccine	5.21 ± 0.19	1.49 ± 0.33
C	sIFITM1 + Vaccine	7.72 ± 0.20***###	1.68 ± 0.23
D	sIFITM3 + Vaccine	7.45 ± 0.39**###	1.27 ± 0.27
E	sViperin+Vaccine	7.94 ± 0.67**##	2.31 ± 0.53

Compared with the control group, ** *p* < 0.01, and *** *p* < 0.001. Compared with the vaccine group, ## *p* < 0.01, ### *p* < 0.001.

#### 3.3.2 As adjuvants sIFITM1, sIFITM3 and sViperin showed good safety

**Figure 6:**
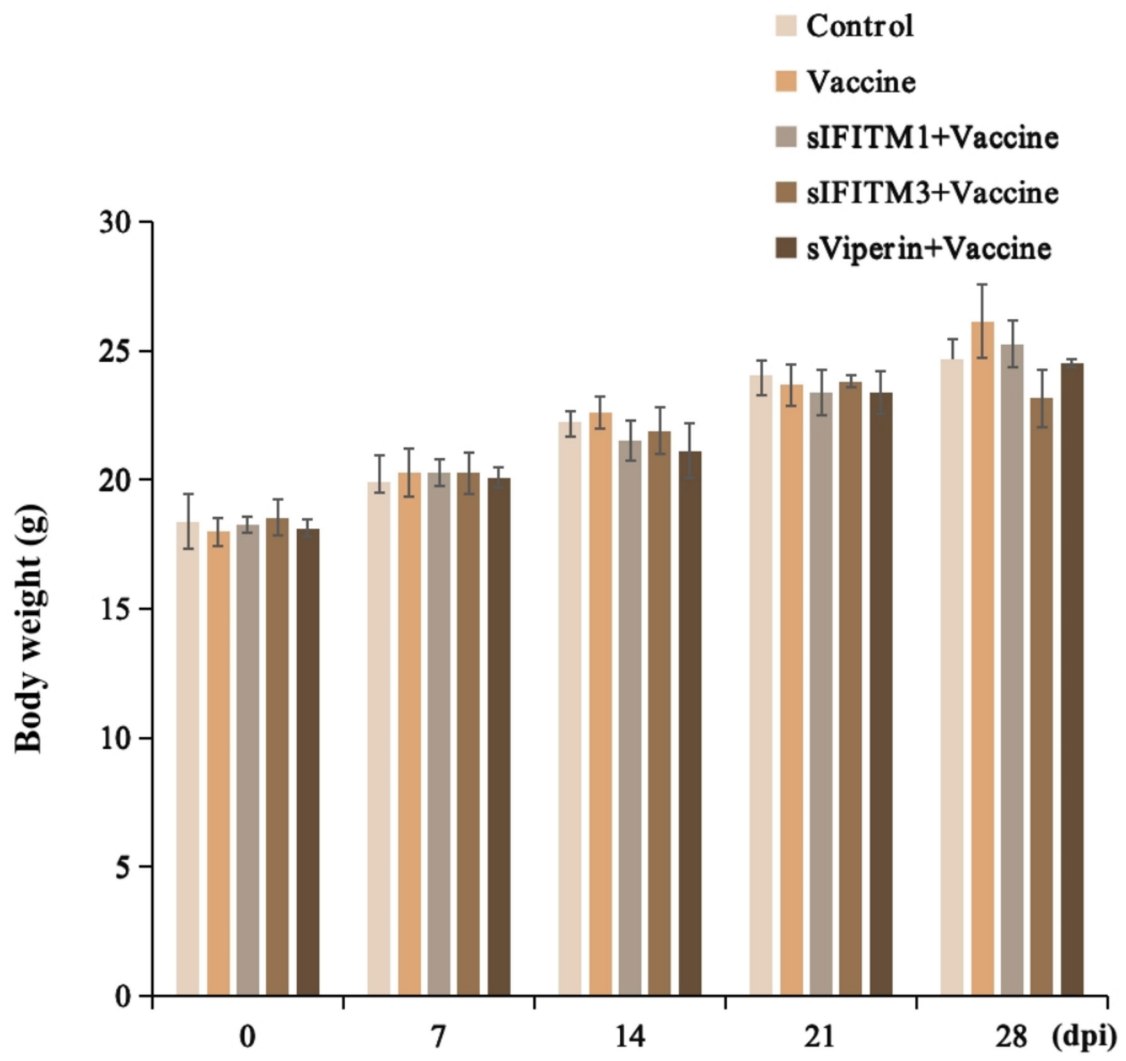
The effects of fusion proteins sIFITM1, sIFITM3 and sViperin on body weight in immunized mice.

The secondary immunization was performed 21 days after the first immunization, during which the weight of mice was weighed every 7 days and the weight changes were detected. The effects of sIFITM1, sIFITM3, and sViperin fusion proteins on body weight of mice were shown in Figure 6. Compared with the control group, all groups gained weight after immunization, but the difference was no significant. Compared with vaccine control group, there were no significant differences in adjuvants with vaccine groups.

**Figure 7:**
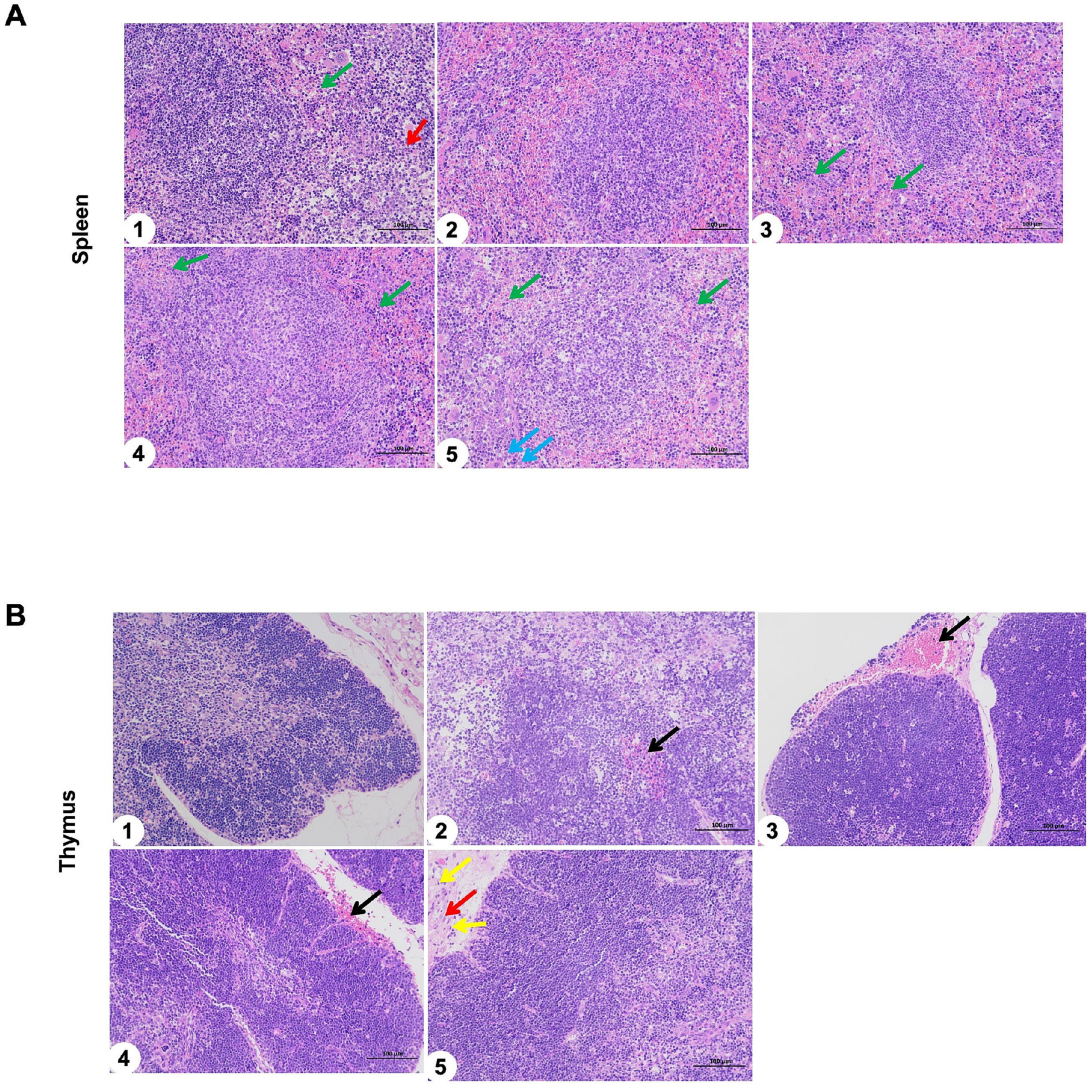
The spleen and thymus of immunized mice were observed with H&E staining. **(A,B)** The effects of sIFITM1, sIFITM3 and sViperin on the spleen **(A)** and thymus **(B)** of immunized mice were analyzed by H&E staining. 1: Control; 2: Vaccine; 3: sIFITM1 + Vaccine; 4: sIFITM3 + Vaccine; 5: sViperin+ Vaccine. A small amount of granulocyte infiltration (green arrow); Extramedullary hematopoietic cells (red arrow); A small number of extramedullary hematopoietic cells around the trabeculae of the spleen (blue arrow); slight bleeding at the local edge (black arrow).

In addition, the spleen and thymus of immunized mice were observed with H&E staining. The effects of sIFITM1, sIFITM3, and sViperin fusion proteins on the spleen of mice were shown in Figure 7A. The results of blank Control group, Vaccine control group, adjuvant and vaccine combined group were the same: normal spleen structure, no obvious abnormality; the red and white pulp is clear, with a small amount of granulocyte infiltration (green arrow) and extramedullary hematopoietic cells (red arrow). Moreover, the effects of sIFITM1, sIFITM3, and sViperin fusion proteins on the thymus of mice were shown in Figure 7B. Compared with the untreated control group, the vaccine group, sIFITM1 + Vaccine group, sIFITM3 + Vaccine group, and sViperin+Vaccine group all exhibited normal thymus architecture, with no significant necrosis observed.

The above results demonstrated that, compared with the blank control group, the spleen and thymus of the vaccine groups (Vaccine, sIFITM1 + Vaccine, sIFITM3 + Vaccine, and sViperin + Vaccine) exhibited no significant pathological damage. This preliminary suggests that sIFITM1, sIFITM3, and sViperin fusion proteins as adjuvants showed good safety.

#### 3.3.3 sIFITM1, sIFITM3, and sViperin significantly increased the expression of IgG and CSFV E2 antibody and the secretion of lymphokines in immunized mice

**Figure 8:**
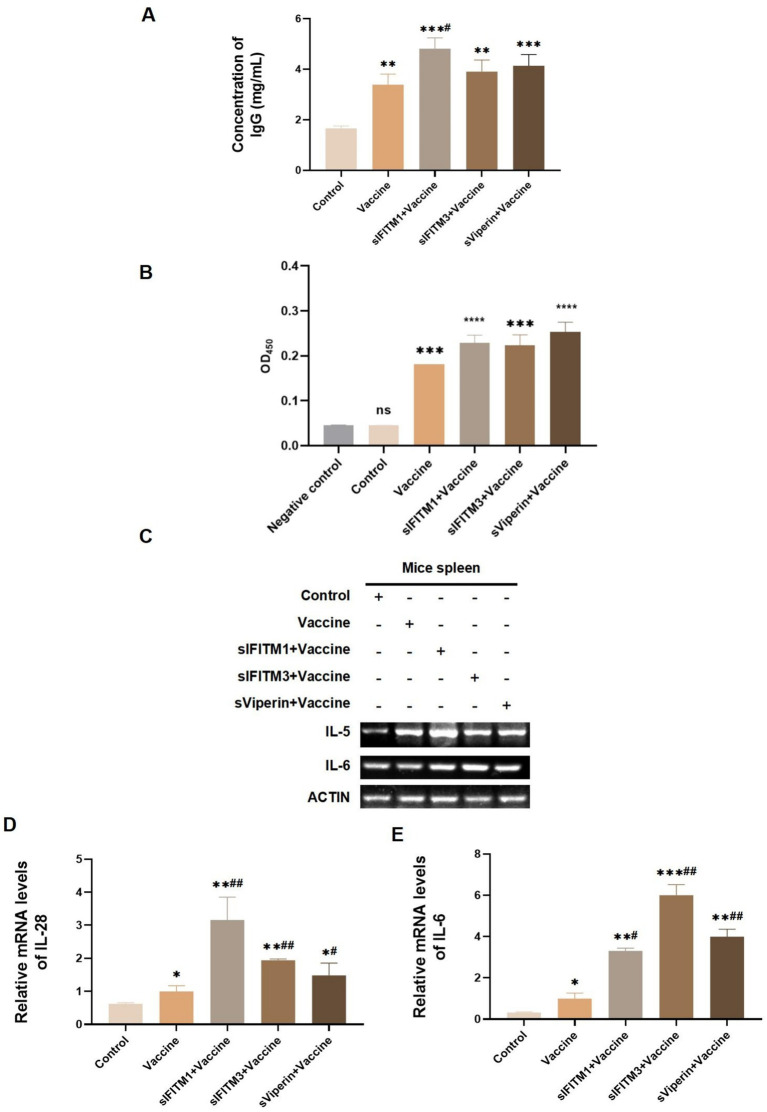
The expression of IgG, CSFV E2 antibody, and lymphokines in immunized mice were analyzed. **(A,B)** On the 7th day after immunization, the blood of mice in each group was collected by eyeball blood collection, serum was separated, and IgG expression level in serum of mice in each group was detected by mouse IgG ELISA kit **(A)** or CSFV E2 antibody ELISA kit **(B)**. Compared with the blank control group, ** means *p* < 0.01, *** means *p* < 0.001; Compared with the vaccine group, # means *p* < 0.05. Negative control is from the ELISA kit and represents a negative sample. **(C–E)** The mRNA level of indicated secretary lymphokines in the spleen of mice in each group was detected by RT-PCR **(C)** and RT-qPCR **(D,E)**. Compared with the blank control group, ** means *p* < 0.01, *** means *p* < 0.001; Compared with the vaccine group, # means *p* < 0.05.

On the 7th day after the secondary immunization, the blood of mice in each group was collected by ocular blood collection. The serum was separated and the protein levels of IgG and specific CSFV E2 antibody in serum of mice in each group was detected. The ELISA assay showed that the IgG was significantly increased in vaccine, sIFITM1 + Vaccine, sIFITM3 + Vaccine, and sViperin+ Vaccine group (*p* < 0.01). Compared with the Vaccine group, IgG was significantly increased in sIFITM1 + Vaccine, sIFITM3 + Vaccine, and sViperin+ Vaccine group (Figure 8A). Consistent with the IgG results, data showed that compared with the Vaccine group, the protein levels of CSFV E2 antibody in the sIFITM1 + Vaccine group was significantly increased. There was also an increasing trend in both the sIFITM3 + Vaccine group and the sViperin+ Vaccine group (Figure 8B). The results suggested that sIFITM1, sIFITM3, and sViperin significantly increased the expression of IgG and specific CSFV E2 antibody in serum of immunized mice.

Th1 cells are a type of helper CD4T cells and mainly secrete IL-28 promotes T cell-mediated immune responses. Th2 cells can secrete cytokines such as IL-4 and IL-5 to enhance antibody-mediated the humoral immune response. The main cytokines Th17 cells executes are IL-17 and IL-6 (9). Therefore, we also detected the secretion of these related cytokines. On the 7th day after the secondary immunization, the secretion of specific immuned-related lymphokines in spleen of mice was detected by RT-qPCR. As shown in Figures 8C–E, the mRNA expressions of IL-5, IL-28, and IL-6 in the vaccine, sIFITM1 + Vaccine, sIFITM3 + Vaccine, and sViperin+ Vaccine group all showed an increasing trend compared with the control group. Compared with the Vaccine group, the mRNA expressions of IL-5, IL-28, and IL-6 in sIFITM1 + Vaccine, sIFITM3 + Vaccine, and sViperin+ Vaccine group were significantly increased (Figures 8C,D). These results indicate that sIFITM1, sIFITM3, and sViperin can enhance the secretion of immuned-related lymphokines, including IL-5, IL-28, and IL-6, in the spleen of immunized mice.

## 4 Discussion

Adjuvants, as an auxiliary substance, can reduce the antigen quantity demand, and enhance the vaccine-induced host immune response to improve vaccine efficiency (49). Adjuvants play crucial roles in improving the efficiency of vaccines, especially in vaccines with weak immunogenicity, such as inactivated vaccines, synthetic peptide vaccines, subunit vaccines, and DNA vaccines.

In recent years, studies have found that there are many ISGs with unique antiviral functions in host cells (22). Taking IFITMs as an example, they can inhibit the membrane fusion reaction between viruses and target cells by changing the sequence of membrane lipids in cells, thereby altering the rigidity and fluidity of membranes (50). sViperin has the ability to catalyze the formation of 3′ -deoxy-3′, 4′ -didehydro-CTP (ddhCTP). ddhCTP can be incorporated into nascent RNA, thereby directly inhibiting viral replication (51). The diversity and specific functions of these ISGs provide host cells with a comprehensive antiviral line of defense. However, the potential capabilities of IFITMs and Viperin as immune enhancers have not been reported in the literature. This means that the application prospect of IFITMs and Viperin in the direction of adjuvant research and development is still a blank area worth further exploration.

We expressed and purified three swine ISGs: sIFITM1, sIFITM3, and sViperin by the *E. coli* expression system, and configured them as vaccine adjuvants. The cells were infected with viruses, and then cultured with sIFITM1, sIFITM3, and sViperin fusion proteins. The data showed that sIFITM1, sIFITM3, and sViperin had certain antiviral activity *in vitro*. After comprehensive consideration of the experimental results, sViperin had the strongest function in resisting swine H3N2 and PRV infection.

We further explored the potential of sIFITM1, sIFITM3, and sViperin as vaccine adjuvants in enhancing immune effects. Inactivated swine fever vaccine is the main means to prevent CSFV, but its immune response is limited, and mice are often used as the model animal of swines in scientific research. To this end, we combined the three fusion proteins with the inactivated recombinant baculovirus vaccine of CSFV E2 protein and applied them to immunize BALB/c mice. Compared with the vaccine group alone, the groups with sIFITM1, sIFITM3, and sViperin fusion proteins showed significant immune-enhancing effects. The organ coefficient and H&E staining of immune mice displayed that sIFITM1, sIFITM3, and sViperin fusion proteins effectively improved the immune response of the spleen, and have a high degree of safety.

ConA is a mitogen of T lymphocytes, which mainly promotes the proliferation of T lymphocytes, and the proliferation ability of T lymphocytes is usually an important indicator of the cellular immunity level (52). In this study, spleen T lymphocytes of immunized mice were stimulated by ConA for 24 h, and the proliferation rate of T lymphocytes increased after sIFITM1, sIFITM3 and sViperin. We found that the sIFITM1, sIFITM3, and sViperin fusion proteins, when combined with inactivated CSFV E2 protein recombinant baculovirus vaccine, can significantly enhance the cellular immune response in mice by promoting the proliferation of splenic T lymphocytes, thereby functioning as effective vaccine immune adjuvants.

IgG is an indicator of humoral immune response (53). When pathogenic microorganisms enter the body, the immune system will be activated and corresponding antibodies against pathogenic microorganisms will be produced. IgG antibody plays the role of activating complement and synthesizing various toxins in immune response (54). Moreover, immune-related factors are important cytokines involved in cellular immune response (55). In this study, we found that sIFITM1, sIFITM3, and sViperin fusion proteins could significantly increase the expression of IgG and CSFV E2 antibodies in serum of immunized mice, and significantly increase the mRNA levels of IL-28, and IL-6 in spleen. The combination of sIFITM1, sIFITM3, and sViperin fusion proteins with inactivated CSFV E2 protein recombinant baculovirus vaccine significantly enhances both cellular and humoral immune responses by increasing the expression of IgG and CSFV E2 antibodies and lymphokines such as IL-5, IL-28, and IL-6. This synergistic effect plays a crucial role in augmenting immunogenicity of the vaccine.

In summary, the combination of sIFITM1, sIFITM3, and sViperin with inactivated CSFV vaccine as adjuvants significantly enhances the vaccine immunogenicity and improves the immune response in vaccinated mice. This study provides a theoretical foundation for advancing the development of animal vaccine adjuvants and identifies promising candidate molecules for the development of novel and highly effective antiviral drugs and vaccine adjuvants.

## 5 Conclusion

In this study, three porcine ISGs, namely IFITM1, IFITM3, and Viperin, were successfully expressed and purified using a prokaryotic expression system. Additionally, based on the Ni-NTA affinity chromatography purification method, an alternative approach involving ammonium sulfate precipitation was explored for protein purification. This method not only enhanced the purification efficiency but also reduced costs, thereby establishing a robust foundation for future large-scale production and industrial application. Furthermore, *in vitro* experiments demonstrated that the fusion proteins sIFITM1, sIFITM3, and sViperin exhibit broad-spectrum antiviral activities. This underscores their critical roles in the antiviral defense mechanism and identifies them as promising candidate molecules for the development of novel antiviral therapeutics.

Simultaneously, these antiviral proteins were evaluated as vaccine adjuvants. Immunization experiments on BALB/c mice have shown that when sIFITM1, sIFITM3, and sViperin fusion proteins are utilized in combination with the vaccine, they effectively enhance the immune protective efficacy of the vaccine by strengthening cellular and humoral immune responses. This discovery underscores the potential of ISGs as effective vaccine immune enhancers, deepens our understanding of their roles in immune regulation, and provides a theoretical basis for optimizing vaccine design and enhancing vaccine efficacy.

## Data Availability

The original contributions presented in the study are included in the article/Supplementary material, further inquiries can be directed to the corresponding author.
