# Supplementary material for: sIFITM1, sIFITM3, and sViperin antiviral proteins as inactivated CSFV vaccine adjuvants
Source: Front Vet Sci. 2025 Aug 26;12:1661103. doi: 10.3389/fvets.2025.1661103 (PMC12417131; doi:10.3389/fvets.2025.1661103)
Supplement: Supplementary file 1 [file Data_Sheet_1.pdf]

Figure S1 The induced expression of fusion proteins sIFITM1, sIFITM3, and sViperin

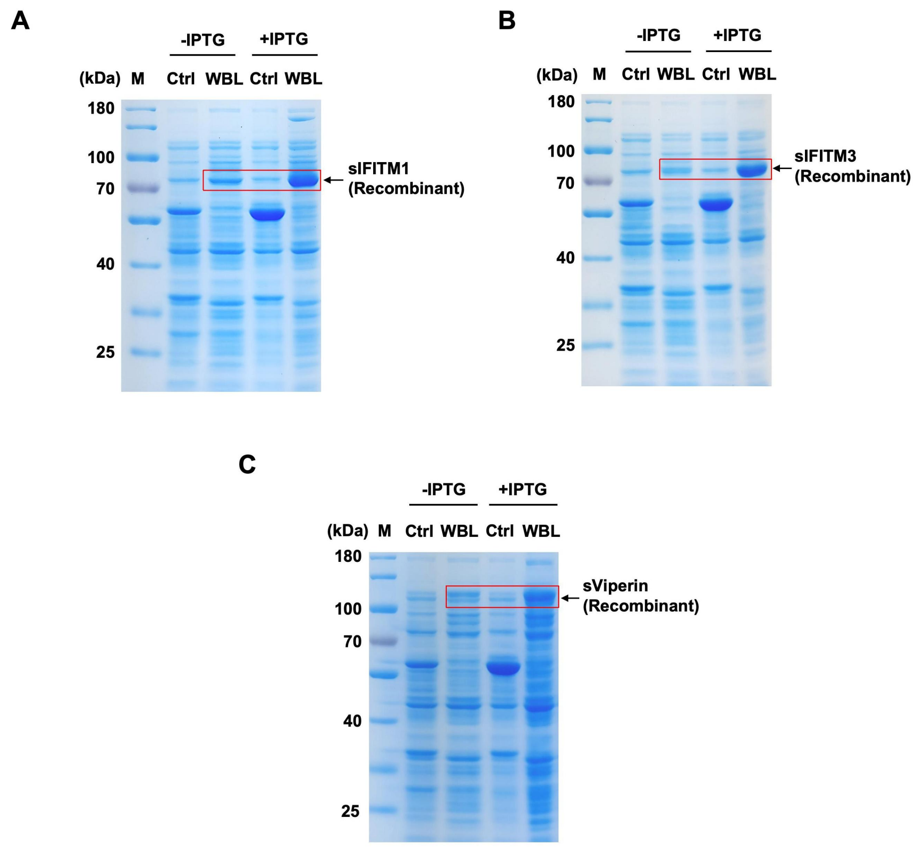

(A-C) The induced expression of fusion proteins sIFITM1, sIFITM3, and sViperin was detected by SDS-PAGE. Expression of sIFITM1 (A), sIFITM3 (B), and sViperin (C).

Figure S2 Fusion proteins sIFITM1, sIFITM3, and sViperin inhibited H3N2 replication in PK-15 cells

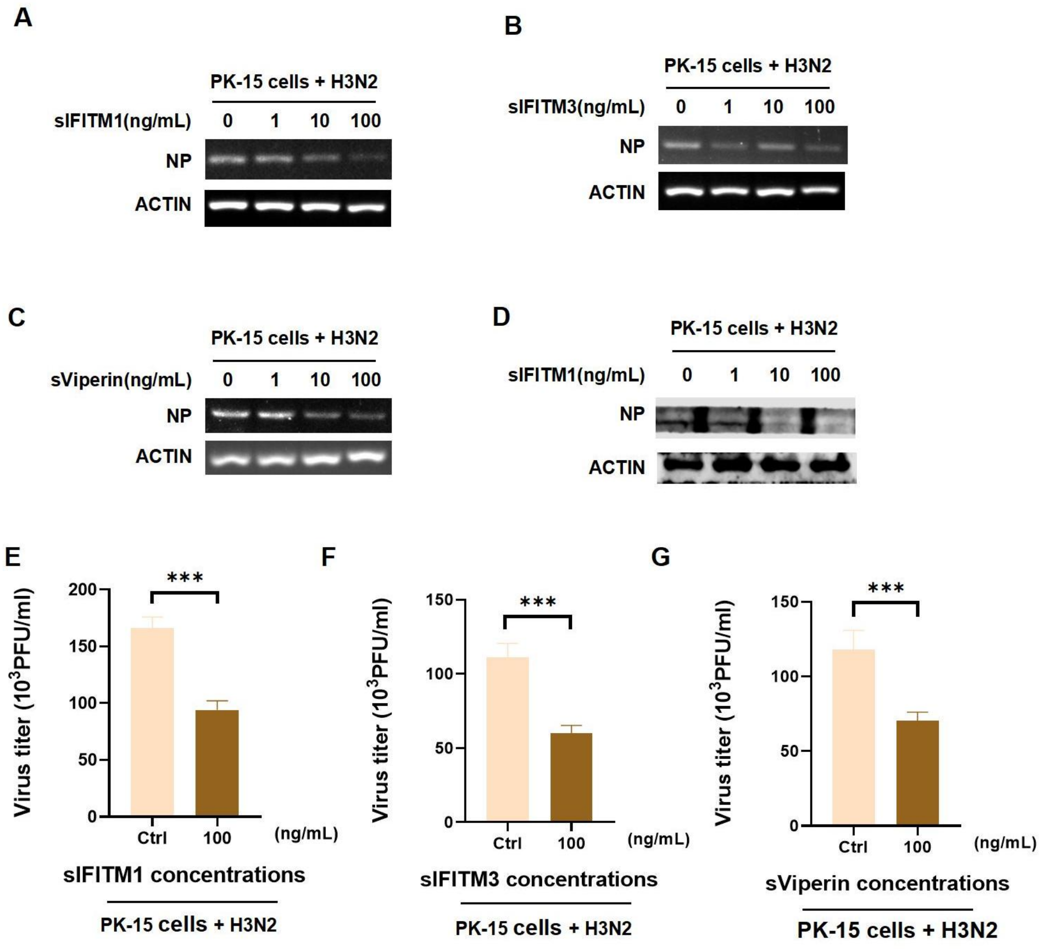

(A-E) Following a 2-hour adsorption period of H3N2 (MOI=1) on PK-15 cells, the fusion proteins sIFITM1, sIFITM3, and sViperin were introduced to cells at indicated concentrations, respectively, for 24 hours. Subsequently, RT-PCR (A-C) and Western blotting (D) were conducted to assess the mRNA or protein levels of H3N2-NP. (E-G) Viral titers of H3N2 in the supernatant were detected by plaque assay. Data are represented as mean ± SD. **P< 0.01; ***P< 0.01.

Table S1　The HPLC analysis result of sIFITM1

Peak No.	Retention time [min]	Peak height [uAU]	Peak area [uAU*s]	Area %	Content %
1	6.127	155	6245.8	1.1584	1.1584
2	7.956	7944.4	190449.5	35.3208	35.3208
3	8.49	5873.2	312338	57.926	57.926
4	11.098	144.1	4918.1	0.9121	0.9121
5	12.08	79.3	2314.1	0.4292	0.4292
6	12.737	220	10032.3	1.8606	1.8606
7	13.899	250.4	12901.3	2.3927	2.3927
total:		14666.4	539199.5	100	100

Table S2　The HPLC analysis result of sIFITM3

Peak No.	Retention time [min]	Peak height [uAU]	Peak area [uAU*s]	Area %	Content %
1	6.111	112	3218.3	0.3854	0.3854
2	7.904	16216.9	386227.6	46.2537	46.2537
3	8.55	6676.2	392088.6	46.9556	46.9556
4	11.151	347.7	12041	1.442	1.442
5	11.934	410.7	19279.1	2.3088	2.3088
6	12.678	371.6	16790.3	2.0108	2.0108
7	13.86	246.3	5375.5	0.6438	0.6438
total:		24381.4	835020.4	100	100

Table S3　The HPLC analysis result of sViperin

Peak No.	Retention time [min]	Peak height [uAU]	Peak area [uAU*s]	Area %	Content %
1	6.127	155	6245.8	1.1584	1.1584
2	7.956	7944.4	190449.5	35.3208	35.3208
3	7.908	2219	2219	57.5046	57.5046
4	11.098	144.1	4918.1	0.9121	0.9121
5	12.08	79.3	2314.1	0.4292	0.4292
total:		3137.4	150571.3	100	100
